# NF-κB/p65 Competes With Peroxisome Proliferator-Activated Receptor Gamma for Transient Receptor Potential Channel 6 in Hypoxia-Induced Human Pulmonary Arterial Smooth Muscle Cells

**DOI:** 10.3389/fcell.2021.656625

**Published:** 2021-12-07

**Authors:** Yan Wang, Naijian Li, Yingfeng Wang, Guobing Zheng, Jing An, Chang Liu, Yajie Wang, Qicai Liu

**Affiliations:** ^1^The First Affiliated Hospital of Guangzhou Medical University, Guangzhou, China; ^2^Department of Cardiology, Laboratory of Heart Center, Zhujiang Hospital of Southern Medical University, Guangzhou, China; ^3^Department of Guangdong Provincial Key Laboratory of Shock and Microcirculation, Guangzhou, China; ^4^Prenatal Diagnosis Unit, Boai Hospital of Zhongshan, Zhongshan, China; ^5^Department of Academic Research Office, Zhujiang Hospital of Southern Medical University, Guangzhou, China; ^6^Department of Scientific Research Center, Southern Medical University, Guangzhou, China; ^7^Dermatology Hospital of Southern Medical University, Guangzhou, China; ^8^Southern Medical University Institute for Global Health and Sexually Transmitted Diseases, Guangzhou, China

**Keywords:** hypoxia, pulmonary artery hypertension, transient receptor potential channel 6, store-operated calcium entry, Ca^2+^ influx

## Abstract

**Objective:** Peroxisome proliferator-activated receptor gamma (PPARγ) has an anti-proliferation effect on pulmonary arterial smooth muscle cells (PASMCs) via the transient receptor potential channel (TRPC) and protects against pulmonary artery hypertension (PAH), whereas nuclear factor-kappa B (NF-κB) has pro-proliferation and pro-inflammation effects, which contributes to PAH. However, the association between them in PAH pathology remains unclear. Therefore, this study aimed to investigate this association and the mechanisms underlying TRPC1/6 signaling-mediated PAH.

**Methods:** Human pulmonary arterial smooth muscle cells (hPASMCs) were transfected with p65 overexpressing (pcDNA-p65) and interfering plasmids (shp65) and incubated in normal and hypoxic conditions (4% O_2_ and 72 h). The effects of hypoxia and p65 expression on cell proliferation, invasion, apoptosis, [Ca^2+^]i, PPARγ, and TRPC1/6 expression were determined using Cell Counting Kit-8 (CCK-8), Transwell, Annexin V/PI, Fura-2/AM, and western blotting, respectively. In addition, the binding of p65 or PPARγ proteins to the TRPC6 promoter was validated using a dual-luciferase report assay, chromatin-immunoprecipitation-polymerase chain reaction (ChIP-PCR), and electrophoretic mobility shift assay (EMSA).

**Results:** Hypoxia inhibited hPASMC apoptosis and promoted cell proliferation and invasion. Furthermore, it increased [Ca^2+^]i and the expression of TRPC1/6, p65, and Bcl-2 proteins. Moreover, pcDNA-p65 had similar effects on hypoxia treatment by increasing TRPC1/6 expression, [Ca^2+^]i, hPASMC proliferation, and invasion. The dual-luciferase report and ChIP-PCR assays revealed three p65 binding sites and two PPARγ binding sites on the promoter region of TRPC6. In addition, hypoxia treatment and shPPARγ promoted the binding of p65 to the TRPC6 promoter, whereas shp65 promoted the binding of PPARγ to the TRPC6 promoter.

**Conclusion:** Competitive binding of NF-κB p65 and PPARγ to TRPC6 produced an anti-PAH effect.

## HIGHLIGHTS

- Hypoxia increased p65 expression, [Ca^2+^], and proliferation in hPASMCs.

- NF-κB/p65 expression was positively correlated with hPASMC proliferation and invasion.

- NF-κB/p65 mediated hPASMC proliferation by promoting TRPC6 expression.

- NF-κB/p65 competed with PPARγ for TRPC6 in hypoxia.

## Introduction

Pulmonary arterial hypertension (PAH) is a pathological condition characterized by increased vascular growth and proliferation, which results in excessive pulmonary vascular remodeling and heart dysfunction (Wang et al., 2015; Rosenzweig et al., 2019). Although the mechanism behind the onset and development of PAH remains unknown, a variety of factors, including increased intracellular Ca^2+^ concentration ([Ca^2+^]i) and hypoxia-induced pathological changes, are known to be associated with PAH pathology (Wang et al., 2006, 2015; Lu et al., 2008; Xu et al., 2014; Zhang Y. et al., 2014).

[Ca^2+^]i is controlled by the store-operated calcium channel (SOCC), which regulates the store-operated Ca^2+^ entry (SOCE) in the human pulmonary arterial smooth muscle cells (hPASMCs) (Wang et al., 2006, 2015; Lu et al., 2008; Xu et al., 2014; Zhang Y. et al., 2014). Our earlier studies had shown that SOCC members, including transient receptor potential canonical (TRPC) proteins 1 and 6, in mammalian cells are responsible for the increased [Ca^2+^]i and PAH (Wang et al., 2006; Lu et al., 2008; Xu et al., 2014; Zhang Y. et al., 2014).

Several studies have found that hypoxic conditions upregulate the expression of TRPC1 and TRPC6, which then promotes SOCE, increases the [Ca^2+^]i, and induces hPASMC proliferation (Wang et al., 2006; Lu et al., 2008; Xu et al., 2014; Zhang Y. et al., 2014). Furthermore, hypoxic conditions suppress the expression of peroxisome proliferator-activated receptor gamma (PPARγ), a therapeutic target for the treatment of PAH (Nisbet et al., 2010; Gong et al., 2011; Wang et al., 2015). A few other research studies confirmed that the therapeutic effect of sildenafil on PAH is associated with the suppression of TRPC1/6 by PPARγ activation (Gong et al., 2011; Wang et al., 2013a; Zhang D. et al., 2014; Liu et al., 2018). We found in our previous experiments that PPARγ inhibits PAH by targeting SOCE and TRPC1/6, which results in anti-proliferation and pro-apoptosis effects on PASMCs (Wang et al., 2015).

There is plenty of evidence showing the antagonistic or competing biological functions of nuclear factor-kappa B (NF-κB) and PPARγ in inflammation, nephrotoxicity, and myocardial ischemia–reperfusion injury (Eisele et al., 2013; Scirpo et al., 2015; Liu et al., 2016; Zhang et al., 2016). Furthermore, NF-κB activation contributes to PAH (Patel et al., 2017; Shi et al., 2018; Zhai et al., 2018). Moreover, NF-κB activation promotes the excessive proliferation and growth of hPASMCs (Han and Logsdon, 2000) and elevates the [Ca^2+^]i via regulating TRPC1 (Yang et al., 2009; Li et al., 2017; Zhao et al., 2019). This indicates that NF-κB plays a crucial role in the development of PAH via regulating SOCE and [Ca^2+^]i. Therefore, we assumed that there might be an association between NF-κB and PPARγ on the regulation of TRPC1/6, SOCE, and the pathogenesis of PAH. However, the interaction of NF-κB and PPARγ with TRPC1/6 as well as with the development of PAH is not clear.

To address this issue, we performed this study to investigate the association of NF-κB/p65 with PPARγ in chronic hypoxia-induced PAH and with TRPC1/6-mediated SOCE. We determined the expression of p65 in the chronic PAH model and its effect on hPASMC proliferation, growth, invasion, [Ca^2+^]i, and TRPC1/6 activity. Furthermore, the molecular correlations of PPARγ and NF-κB p65 with PAH development under hypoxic conditions are investigated.

## Materials and Methods

### Cells and Culture Conditions

The hPASMCs were obtained from ScienCell (Carlsbad, CA, United States). Cells were incubated in smooth muscle growth medium-2 (SmGM-2, ScienCell) supplemented with 5% fetal calf serum and growth factors at 37°C with 5% CO_2_ as previously described (Schultz et al., 2007).

### Hypoxia Treatment of Cells

The *in vitro* cellular PAH model was established by treating hPASMCs with hypoxic conditions. hPASMCs were seeded into a 24-well plate and incubated for 24 h at 37°C in 5% CO_2_. After forming a monolayer, hPASMCs were incubated in fresh SmGM-2 for 72 h at 37°C in 4% O_2_. Each experiment was replicated three times.

### Plasmid Construction and Cell Transfection

pcDNA-p65 and pcDNA-PPARγ plasmids were constructed by cloning the complete p65 and PPARγ genes into pcDNA3.0 vectors (GenePharma Co., Ltd., Shanghai, China) using double-restriction enzyme (*Kpn*I and *Xho*I, Thermo Fisher Scientific Inc., Waltham, MA, United States) and T4 ligase (Thermo Fisher Scientific Inc.). The shRNAs and scrambled sequences targeting p65 (shp65) and PPARγ (shPPARγ) were purchased from GenePharma Co., Ltd. The cells were transfected using the Lipofectamine 2000 transfection reagent (Invitrogen, United States) before exposing them to hypoxic conditions. This experiment was conducted in triplicate according to the usage instructions. At 48 h post transfection, hPASMCs were incubated in SmGM-2 for 72 h at 37°C under normal or hypoxic conditions (4% O_2_).

### Cell Viability Assay

Cell viability was evaluated using the Cell Counting Kit-8 (CCK-8) assay (Beyotime Institute of Biotechnology, Shanghai, China) and the BrdU assay kit (Beyotime). The control and transfected hPASMCs were seeded in 96-well plates (1 × 10^5^ cells per well) and incubated for 72 h at 37°C under normal or hypoxic conditions. For the CCK-8 assay, the cells were harvested, washed, and incubated with CCK-8 solution (10 μl per well) for 1 h. A Bio-Rad microplate reader (Bio-Rad, Hercules, CA, United States) was used to determine the optical density at 450 nm (OD_450 nm_). For the BrdU assay, the control and transfected hPASMCs were seeded in 96-well plates (1 × 10^5^ cells per well) and incubated in normal or hypoxic conditions for 24 h. The BrdU solution was added to a final concentration of 10 μg/ml, and cells were incubated for 48 h. After incubation, the cell slides were prepared by fixing, washing, and incubating cells with HCl (2 mol/L, for 5 min), sodium borate (0.1 mol/L, pH 8.3, for 10 min), and Triton X-100 (0.2%, for 10 min). Subsequently, these cell slides were incubated with 3% bovine serum albumin (BSA) for 1 h, BrdU antibody (1: 200) at 4°C overnight, and secondary IgG/Alexa Fluor 594 (1:100) in the dark for 1 h. DAPI was used for nuclear staining (dark, for 10 min). These stained cells were observed under an Olympus BX51 immunofluorescence microscope (Olympus, Tokyo, Japan).

### *In vitro* Transwell Assay

We determined the influence of hypoxia condition and the expression of p65 and PPARγ on the invasion ability of hPASMCs using the Matrigel-coated Transwell chambers (Corning, NY, United States). In summary, hPASMCs (1 × 10^4^ cells per well) were placed into the upper chambers filled with serum-free medium, while the bottom chambers were filled with completed medium (10% FBS). The chambers were incubated in normal and hypoxic conditions for 72 h at 37°C in 5% CO_2_. Following that, cells adhering to the undersurfaces of the membranes were fixed, washed, and stained with crystal violet. These cells were photographed digitally at five randomly selected fields using an Olympus microscope (Olympus).

### Analysis of Cell Apoptosis

The percentage of apoptotic hPASMCs in response to the aforementioned treatments was assessed using the Annexin V/PI flow cytometric analysis (BD Biosciences, Franklin Lakes, NJ, United States) and TUNEL assay. For this, the blank or transfected hPASMCs were cultured in 24-well plates (1 × 10^5^ cells per well) for 72 h under normal or hypoxic conditions. Then, the cells were harvested, fixed, and stained using the Annexin V/PI solution (10 μl). A BD FACSCalibur flow cytometry (BD Biosciences) was used to determine the percentage of cell apoptosis. For the TUNEL assay, cell slides were fixed, washed, and incubated in PBS containing 2% hydrogen peroxide. After washing with PBS, these cells were treated with the buffer and reaction liquid of TdT (at 37°C for 1 h), DAB solutions (0.05%, for 5 min), methyl green (for 10 min), and xylene following the manufacturer’s instructions. Photographs of these cells were taken using an Olympus microscope (Olympus). We performed all the experiments in triplicate.

### Assessment of Intracellular Calcium Level ([Ca^2+^]i)

Using the Fura-2/AM fluorescence assay kit (Nanjing Jiancheng Bioengineering Institute, Nanjing, China), we evaluated the effect of the hypoxic condition as well as the expression of the p65 and PPARγ genes on the [Ca^2+^]i in hPASMCs. For this, the cells were transfected with shRNA- or pcDNA-expressing plasmids for 48 h and then incubated for 72 h under hypoxic or normal conditions. Then, all the cells were incubated for 1 h in a calcium-free medium containing Fura-2/AM. We used a microplate reader to determine the intracellular calcium levels by measuring the Ex/Em = 380/510 nm and Ex/Em = 340/510 nm values.

### Western Blot Analysis

We extracted cell proteins from hPASMCs using the lysis buffer (Beyotime) and nuclear protein using a Nuclear Protein Extraction Kit Beyotime according to the manufacturer’s instructions. Protein samples were separated using the 10% SDS-PAGE (Invitrogen) and electrotransferred onto PVDF membranes (Millipore, Billerica, MA, United States). The specific primary antibodies against p65 (1:1,000, Abcam), p-p65 (1:1,000), PPARγ (1:1,000), p-PPARγ (1:500), TRPC1 (1:2,000), TRPC6 (1:2,000), Bax (1:2,000), Bcl-2 (1:2,000), GAPDH (1:10,000), and Histone H3 (1:500) were used for primary incubation, and anti-rabbit/rat IgG secondary antibodies (1:20,000, Boster Biotechnology, Wuhan, China) were used for secondary incubation. The optical density of the protein band was measured using the Image-Pro Plus 6.0 software (Media Cybernetics Inc., Bethesda, MD, United States).

### Immunofluorescence Detection

The immunofluorescence technique was used to locate and measure the cytoplasmic p65 and nuclear p-p65 proteins. The control hPASMCs or cells transfected with or without pcDNA-p65 or shp65 plasmids were placed in 24-well plates with slides and incubated for 72 h under normal or hypoxic conditions. Afterward, the cell slides were harvested, fixed, and used for the immunofluorescence assay of p65. Then, a Triton X-100 (1 ml of 0.5%) solution was added into each well, and plates were incubated for 15 min, followed by blocking with 5% BSA for 60 min. The cells were then incubated with anti-p65 (1:500, Abcam) at 4°C overnight, followed by incubation with Alexa Fluor 488-conjugated secondary antibody (1:1,000, Abcam) for 60 min at room temperature and DAPI staining for 10 min in the dark (Beyotime). The immunofluorescence was detected using an Olympus immunofluorescence microscope (Olympus).

### Dual-Luciferase Reporter Assay

We predicted three binding sites of p65 on the promoter region of the TRPC6 gene using Genomatix^1^. The interaction of p65 protein with the promoter region of the TRPC6 gene was detected using the dual-luciferase reporter assay. The pcDNA-p65 plasmid, as well as the plasmid expressing wild-type (WT) and mutant (MUT) TRPC6 promoter sequences, was constructed using the pGL luciferase reporter vectors (GenePharma Co., Ltd.). The plasmids expressing the p65 and TRPC6-WT or TRPC6-MUT sequences were co-transfected into the hPASMCs. We evaluated relative luciferase activity using the Dual-Glo Luciferase Assay system (Promega). Each experiment was performed in triplicate.

### Electrophoretic Mobility Shift Assay

Electrophoretic mobility shift assay was used to confirm the binding of p65 to the promoter of TRPC6. To perform this assay, the nuclear protein was isolated using the methods described before. EMSA was performed using a LightShift Chemiluminescent EMSA Kit (Thermo Fisher Scientific Inc.) according to the manufacturer’s instructions. To summarize, the probes were diluted (1:100, in ddH_2_O) and added to the reaction mixture without or with 5 μg nucleoprotein. Competitor, mutant competitor, and P65 antibody were added separately. After the probes were transferred onto 6.5% polyacrylamide (Genview, Beijing, China) and PVDF membranes (Millipore), a chromogenic reaction was performed. A Microtek Bio-5000 scanner (Microtek Lab, Inc., Santa Fe Springs, CA, United States) and Image-Pro Plus 6.0 software (Media Cybernetics Inc., Bethesda, MD, United States) were used for the analysis. Each reaction was carried out in triplicate.

### Chromatin-Immunoprecipitation

Chromatin-immunoprecipitation assay coupled with quantitative PCR (ChIP-qPCR) was conducted to validate the binding ability of PPARγ and p65 to the promoter region of the TRPC6 gene. The ChIP assay was performed according to the manufacturer’s protocol using a Simple ChIP Plus Enzymatic ChIP Kit (Cell Signaling Technology). For chromatin digestion, hPASMCs were treated with a protease inhibitor cocktail (PIC). Next, ChIP was performed using the protein G magnetic beads (Invitrogen), enriching anti-TRPC6 antibody or IgG (negative control) at 4°C overnight. After elution, the DNA–protein complexes were decrosslinked using proteinase K (CST) at 65°C for 2 h. Then, the resulting DNA fragments were purified and used for the PCR analysis and agarose gel separation (2%, Biowest, Nuaillé, France). We performed all reactions in triplicate. The primers used for ChIP-PCR are shown in Table 1.

**TABLE 1:** The PCR primer sequences used in this study.

**Genes**	**ChIP anti-body**	**Primers**	**Sequences (5′-3′)**
TRPC6	p65	ChIP-PCR primer 1 forward	AAACAGCTTGGAAACGTG
		ChIP-PCR primer 1 reverse	TGATACCGATAAAGAGGC
		ChIP-PCR primer 2 forward	CCCTTAAGTGGTGACTTTTCCC
		ChIP-PCR primer 2 reverse	AGGGGACGACGGTGAAGCA
TRPC6	PPARγ	ChIP-PCR primer 1 forward	TTGAAACGCAGTTGGCAT
		ChIP-PCR primer 1 reverse	CAAGCTGTTTTATTTTTAAGACTT
		ChIP-PCR primer 2 forward	CGCTCTTACGCTTCGCTAC
		ChIP-PCR primer 2 reverse	AGGGGGTGCAAAGAGGATC

### Quantitative Real-Time PCR

The relative expression levels of genes included in this study were measured using real-time quantitative PCR (qRT-PCR). First, total RNA was extracted using the TRIzol reagent (TaKaRa, Tokyo, Japan). Furthermore, separation of cytoplasmic and nuclear RNA was done using the PARIS Kit (Invitrogen) following the manufacturer’s instructions. Moreover, cDNA was synthesized using the Bestar qPCR RT Kit (DBI Bioscience, Shanghai, China). Then, the relative mRNA expression levels of p65, TRPC6, and TRPC1 were determined using a Bestar SYBR Green qPCR Master Mix (DBI Bioscience). Table 1 lists the PCR primer pairs used in this study. We amplified the cDNA on an Agilent Stratagene Mx3000P RT-PCR machine (Agilent, Santa Clara, CA, United States) using the following the reaction procedures: 94°C for 2 min, 40 cycles of 94°C for 20 s, 58°C for 20 s, and 72°C for 20 s. After PCR, relative expression levels of genes were calculated using the standard 2^–ΔΔCt^ methods. β-Actin or GAPDH were used as internal reference genes.

### Statistical Analysis

We used GraphPad Prism 8.0 (GraphPad Software, San Diego, CA, United States) for statistical analyses. The experimental data were expressed as mean ± standard deviation from triplicate. The unpaired *t*-test was used to analyze the differences between two groups and one-way ANOVA to analyze the differences among more than three groups. A *p*-value < 0.05 was considered statistically significant.

## Results

### Hypoxia Increases the Expression of Cytoplasmic and Nuclear p65 Proteins

**FIGURE 1:**
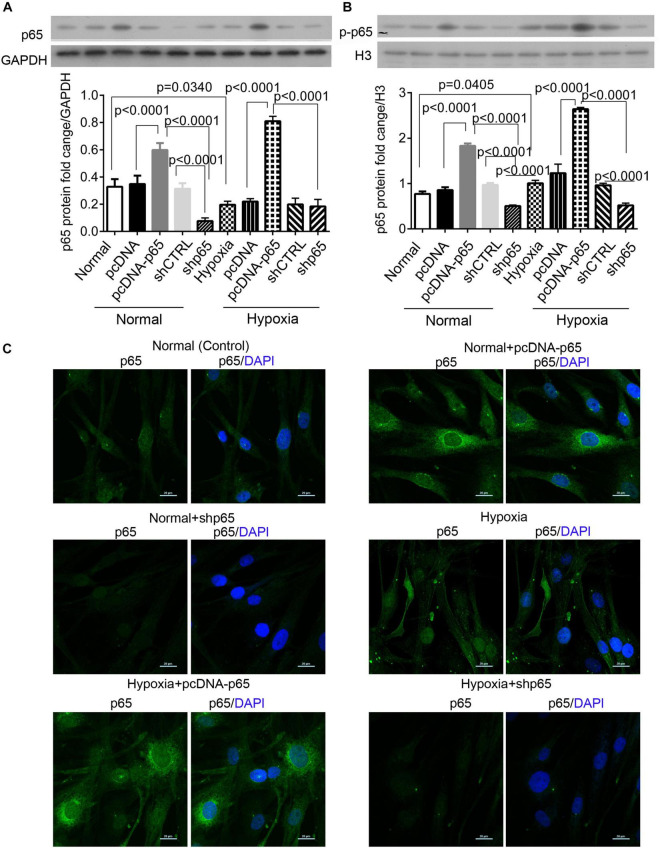
The expression of cytoplasmic and nuclear p65 proteins in response to hypoxia. **(A,B)** The expression levels of the cytoplasmic and nuclear p65 proteins in the hPASMCs upon hypoxia treatment (72 h) with and without the transfections of pcDNA-p65 and shp65. Each experiment was repeated three times. The differences were analyzed using one-way ANOVA. CTRL, control. **(C)** The immunofluorescence assay of the cytoplasmic p65 protein in the hPASMCs upon the above treatments.

We found that hypoxia decreased the expression level of cytoplasmic p65 protein in hPASMCs (*p* = 0.0340, Figure 1A) but significantly upregulated the expression of nuclear p-p65 protein compared with control (*p* = 0.0405, Figure 1B). The pcDNA-p65 transfection increased the levels of the cytoplasmic and nuclear p65 proteins in hPASMCs under both normal and hypoxic conditions (*p* < 0.0001, Figures 1A–C). On the other hand, shp65 transfection decreased the expression levels of the nuclear p65 protein in both normal and hypoxic conditions (*p* < 0.0001, Figure 1B); however, it only decreased the cytoplasmic p65 protein level in the normal condition (*p* < 0.0001, Figure 1B). Moreover, the immunofluorescence assay detected increased the intensity of cytoplasmic p65 protein for hypoxia and pcDNA-p65 transfection but decreased intensity of these proteins for shp65 transfection (Figure 1C). These findings showed that both the cytoplasmic and nuclear p65 proteins were hypoxia inducible.

### p65 Expression Promotes Proliferation and Invasion of Human Pulmonary Arterial Smooth Muscle Cells

**FIGURE 2:**
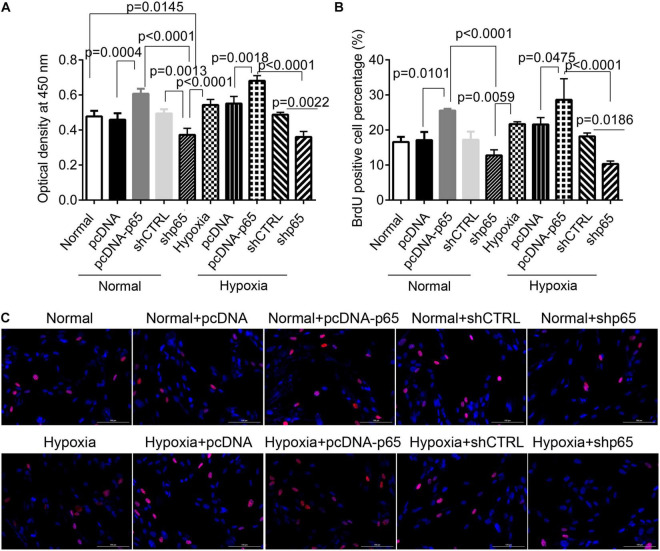
The effect of p65 expression on hPASMC proliferation. **(A)** The cell viability of hPASMC following pcDNA-p65, shp65, and hypoxia using the CCK-8 assay kit. Each experiment was repeated three times. **(B)** The percentage of BrdU-positive hPASMCs following different treatments. The differences were analyzed using one-way ANOVA. **(C)** The representative images of BrdU-positive cells following different treatments. CTRL, control.

The CCK-8 assay revealed that the transfection of pcDNA-p65 significantly increased the proliferation of hPASMCs compared with normal control (*p* = 0.0004, Figure 2A). Similar results were observed in the hPASMCs in hypoxic conditions (*p* = 0.0018, Figure 2A). Inhibiting p65 with shRNA significantly reduced cell viability in both hypoxic (*p* = 0.0022, Figure 2A) and normal conditions (*p* = 0.0013). The results of the BrdU assay were comparable to those of the CCK-8 assay (Figures 2B,C). After pcDNA-p65 transfection, the percentage of BrdU-positive hPASMCs was increased in the normal (*p* = 0.0101) and hypoxic conditions (*p* = 0.0475). Following the shp65 transfection, the percentage of BrdU-positive hPASMCs was downregulated in the hypoxic conditions (*p* = 0.0186, Figure 2B) but not in the normal conditions (*p* > 0.05).

**FIGURE 3:**
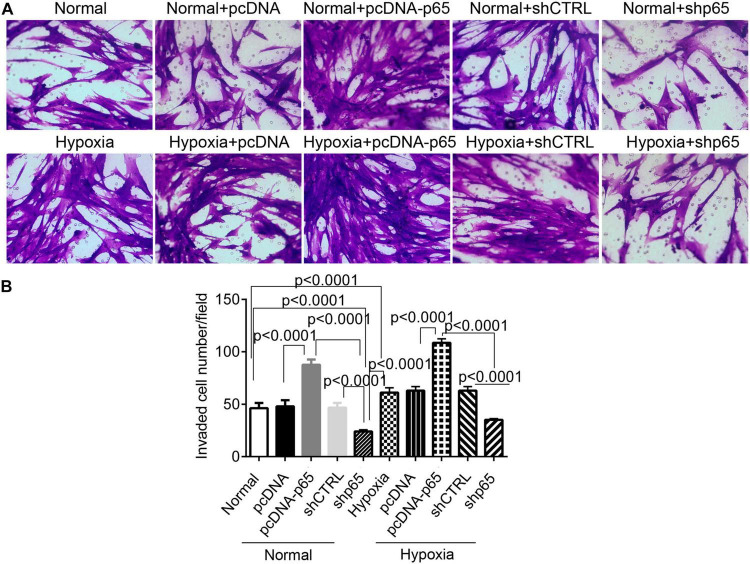
The invasion ability of hPASMCs in response to p65 expression and hypoxia treatment. **(A)** The representative images of the invaded hPASMCs following pcDNA-p65, shp65, and hypoxia treatments. **(B)** The statistical analysis of the invaded cells. The differences were analyzed using one-way ANOVA. CTRL, control.

Furthermore, we found that the number of invaded hPASMCs was increased by pcDNA-p65 (Figures 3A,B). Transfection with pcDNA-p65 increased the number of invaded cells in either the normal (*p* < 0.0001) or hypoxic conditions (*p* < 0.0001; Figures 3A,B), whereas transfection with shp65 decreased the number of invaded hPASMCs in both conditions. These results showed that the expression of p65 promoted cell proliferation and invasion in hPASMCs.

### Hypoxia and p65 Expression Suppress Human Pulmonary Arterial Smooth Muscle Cell Apoptosis

**FIGURE 4:**
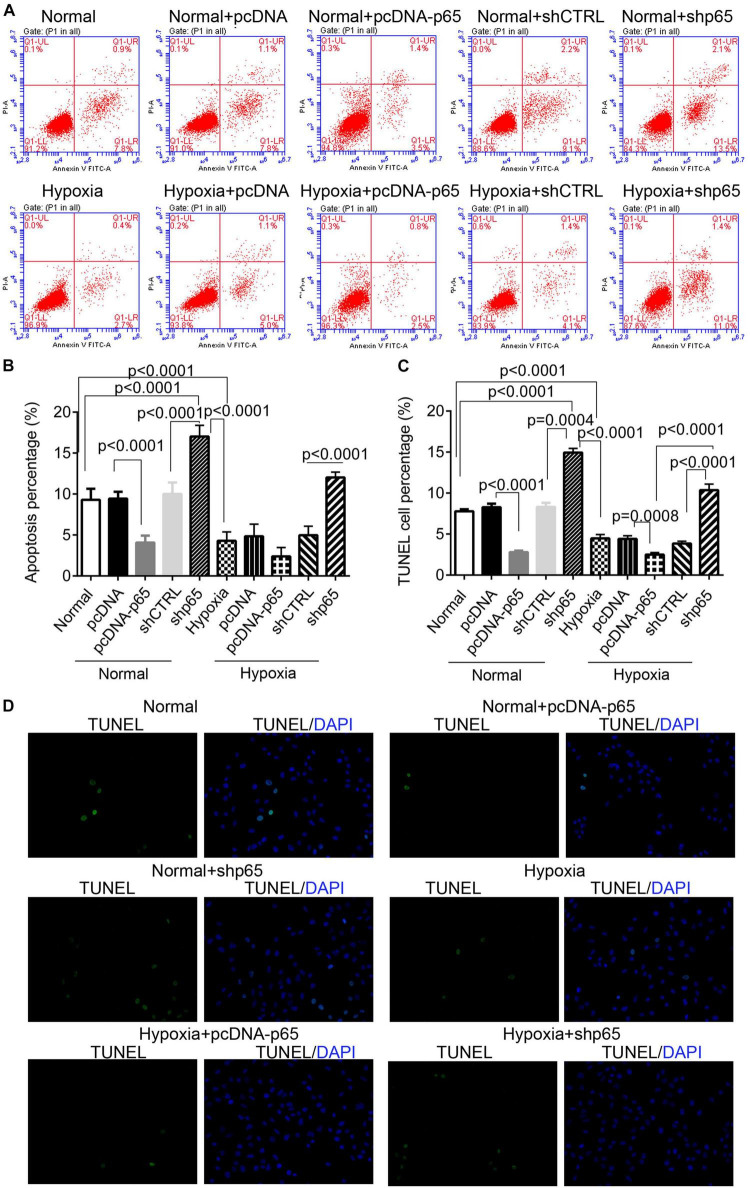
The apoptotic hPASMCs in response to p65 expression and hypoxia treatment. **(A,B)** The representative images and statistical analysis of the apoptotic percentage in hPASMCs using the flow cytometric analysis. Each experiment was repeated three times. **(C,D)** The statistical analysis and representative images of the TUNEL assay in hPASMCs. The differences were analyzed using one-way ANOVA. CTRL, control.

The effect of hypoxia and p65 overexpression on the apoptosis of hPASMCs was confirmed using the flow cytometric analysis and TUNEL assay (Figures 4A–D). Flow cytometry and TUNEL assay indicated that both p65 overexpression (*p* < 0.0001) and hypoxia (*p* < 0.0001) decreased the percentage of apoptotic hPASMCs compared with controls (Figures 4B,C). Furthermore, the inhibition of p65 in hPASMCs increased the apoptotic percentage of hPASMCs in either the normal or hypoxic conditions (*p* < 0.001, Figures 4B,C). These results demonstrated that both hypoxia and p65 expression suppressed the apoptotic percentage of hPASMCs.

### Hypoxia and p65 Expression Promote Ca^2+^ Influx in Human Pulmonary Arterial Smooth Muscle Cells

**FIGURE 5:**
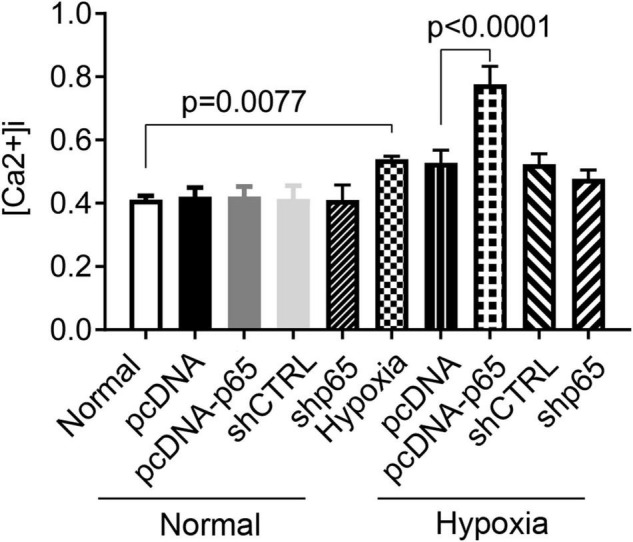
Influence of hypoxia and the expression of p65 on the Ca^2+^ influx. The differences were analyzed using one-way ANOVA. CTRL, control.

As shown in Figure 5, the [Ca^2+^]i in hPASMCs was significantly increased under hypoxic conditions (*p* = 0.0077). Besides, the overexpression of p65 increased the level of [Ca^2+^]i (*p* < 0.0001 for hypoxia + pcDNA-p65 versus hypoxia + pcDNA) in hPASMCs under hypoxic conditions but not in hPASMCs under normal conditions. We also found that the suppression of p65 did not influence the [Ca^2+^]i in either normal or hypoxic conditions.

### The Expression of Related Proteins in Response to Hypoxia and p65 Expression

**FIGURE 6:**
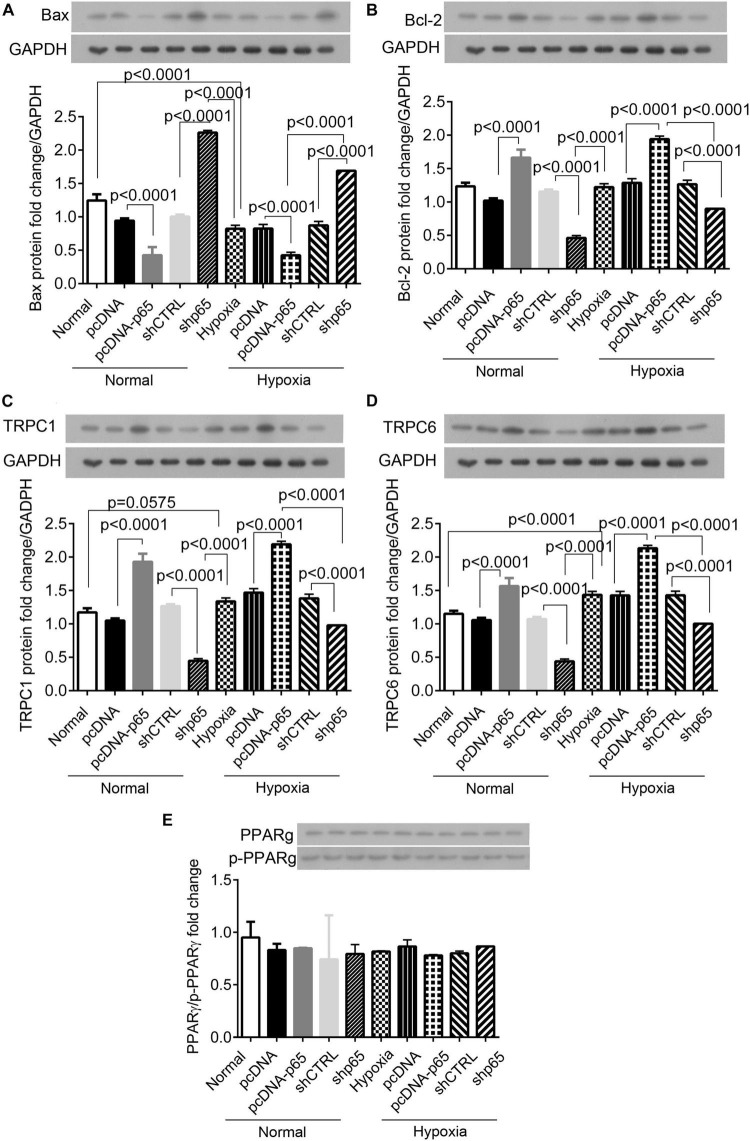
The expression of Bax, Bcl-2, TRPC1, TRPC6, and PPARγ proteins in the hPASMCs. **(A,B)** The expression of Bax and Bcl-2 proteins in the hPASMCs in response to hypoxia, pcDNA-p65, and shp65 treatments. **(C,D)** The expression of the TRPC1 and TRPC6 proteins. **(E)** The fold change of the p-PPARγ to total PPARγ. Each experiment was repeated three times. The differences were analyzed using one-way ANOVA. CTRL, control.

Western blot analysis showed that the expression of apoptosis-related protein Bax in the hPASMCs was upregulated by shp65 transfections (*p* < 0.0001 in normal and hypoxic conditions, Figure 6A). In contrast, the overexpression of p65 downregulated Bax (*p* < 0.0001 in normal conditions and hypoxic conditions, Figure 6A) compared with controls. Furthermore, the expression profile of Bcl-2 was similar to that of the Bax (Figure 6B) but was consistent with the cell proliferation ability in the hPASMCs. The pcDNA-p65 transfection increased Bcl-2 expression (*p* < 0.0001, Figure 6B), whereas shp65 transfection decreased Bcl-2 expression compared with normal controls (*p* < 0.0001, Figure 6B). The hypoxia treatment also decreased Bax (*p* < 0.0001) and increased Bcl-2 (*p* < 0.0001) compared with normal controls.

We also identified the identical expression profiles of the TRPC1 and TRPC6 genes in response to the p65 expression profiling (Figures 6C,D). Overexpression of p65 increased the expression levels of TRPC1 and TRPC6 proteins (*p* < 0.0001 for both normal and hypoxic conditions; Figures 6C,D), whereas shp65 transfection decreased both (*p* < 0.0001; Figures 6C,D). These findings indicated that the expression of TRPC1/6 was inducible by p65. However, this study demonstrated that p65 and hypoxia treatment did not affect the expression of PPARγ or p-PPARγ in the hPASMCs (Figure 6E).

### The Interaction of p65 With the Promoter of TRPC6

**FIGURE 7:**
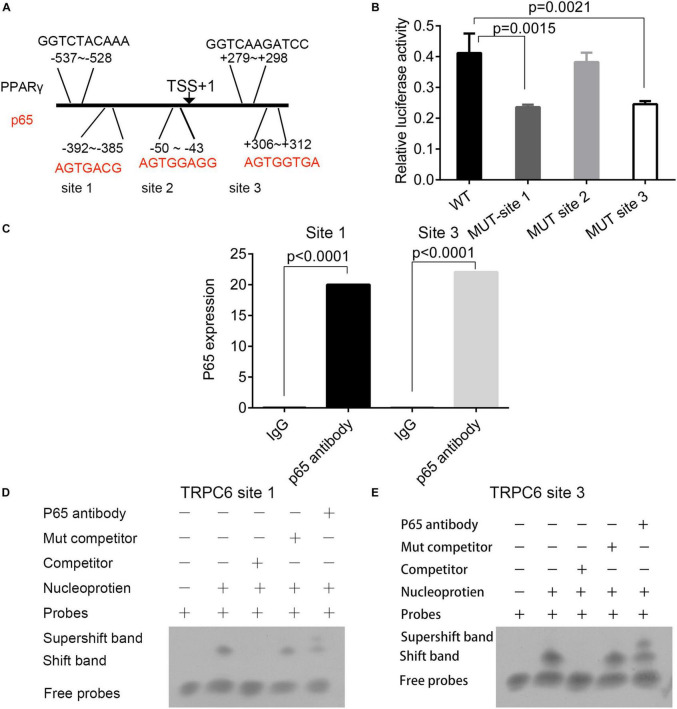
The prediction and validation of p65 protein binding to the promoter region of TRPC6. **(A)** The predicted binding sites of the p65 and PPARγ proteins in the promoter regions of the TRPC6 gene. **(B)** The validation of the binding of the p65 protein on the three sites in the promoter of the TRPC6 gene using the dual-luciferase reporter assay. **(C)** ChIP confirmed the binding of p65 antibodies to the site 1 and site 3 regions on the promoter region of the TRPC6 gene. **(D,E)** EMSA for the binding of the p65 antibody on the two sites. The differences were analyzed using one-way ANOVA. CTRL, control.

Three p65 and two PPARγ binding sites were predicted on the promoter region of the TRPC6 gene (Figure 7A). The interactions of p65 protein with the site 1 (AGTGACG) and site 3 (AGTGGTGA) regions were verified using the dual-luciferase reporter assay, as the relative luciferase activity was decreased following the addition of vectors containing the mutant site 1 (*p* = 0.0015) and 3 sequences (*p* = 0.0021, Figure 7B). ChIP and EMSA also confirmed the binding of p65 antibodies to the site 1 and site 3 regions on the promoter region of the TRPC6 gene (Figures 7C,D). EMSA showed that the addition of the p65 antibody increased the expression of supershift band binding to sites 1 and 3 (Figure 7D).

### The Effect of Hypoxia on the Binding of p65 and Peroxisome Proliferator-Activated Receptor Gamma Proteins on the Promoter of TRPC6

**FIGURE 8:**
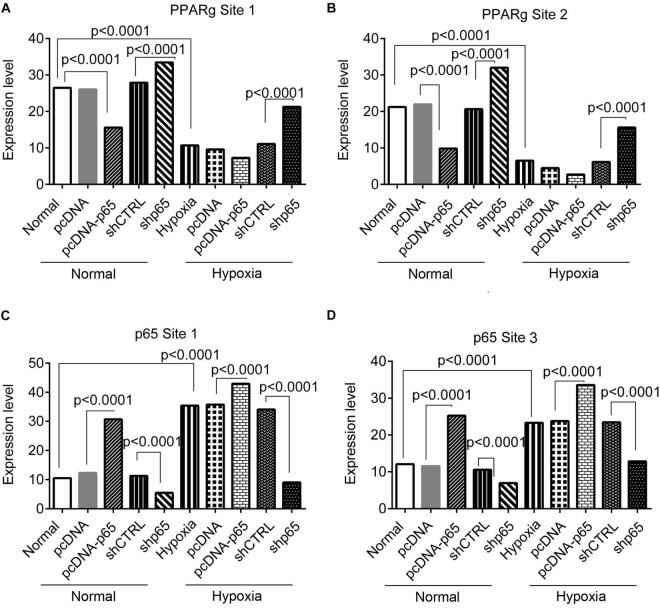
The ChIP-PCR analysis for the binding of the p65 and Par proteins in the promoter regions of the TRPC6 gene in response to p65 overexpression or inhibition. **(A,B)** The PCR results of the DNA fragments including the promoter regions of the TRPC6 gene (site 1 and site 2 in Figure 7) enriched by the PPARγ antibody. **(C,D)** The PCR results of the DNA fragments, including the promoter regions of the TRPC6 gene (site 1 and site 3 in Figure 7) enriched by the p65 antibody. Each experiment was repeated three times. The differences were analyzed using one-way ANOVA. CTRL, control.

**FIGURE 9:**
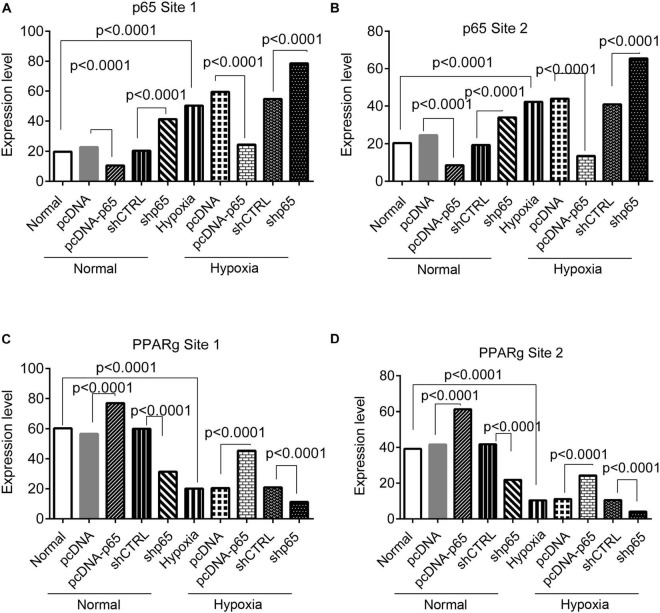
The ChIP-PCR analysis for the binding of the p65 and PPARγ proteins in the promoter regions of the TRPC6 gene in response to PPARγ overexpression or inhibition. **(A,B)** The PCR results of the DNA fragments, including the promoter regions of the TRPC6 gene (site 1 and site 2 in Figure 7) enriched by the p65 antibody. **(C,D)** The PCR results of the DNA fragments, including the promoter regions of the TRPC6 gene (site 1 and site 3 in Figure 7) enriched by the PPARγ antibody. Each experiment was repeated three times. The differences were analyzed using one-way ANOVA. CTRL, control.

**FIGURE 10:**
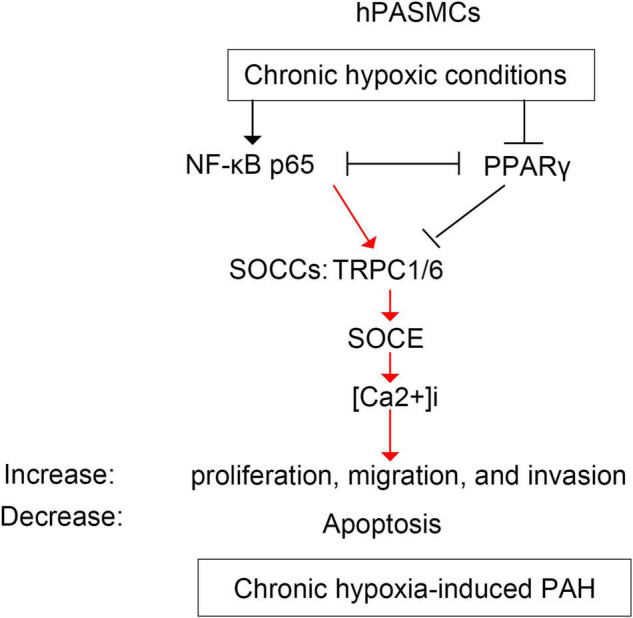
The schematic diagram illustrating the regulation of p65 and PPARγ on TRPC6-mediated SOCE and PAH. hPASMCs, human pulmonary arterial smooth muscle cells; PAH, pulmonary artery hypertension; SOCE, store-operated calcium entry; TRPC, transient receptor potential channel.

The bindings of p65 and PPARγ proteins to the TRPC6 promoter were confirmed using the ChIP-PCR analysis (Figures 8, 9). We found that p65 overexpression or hypoxic conditions promoted the binding of the p65 to the TRPC6 promoter (*p* < 0.0001, at sites 1 and 3) and decreased the binding of the PPARγ to the TRPC6 promoter (*p* < 0.0001, at sites 1 and 2; Figures 8A–D). On the other hand, the inhibition of p65 promoted the binding of PPARγ to the TRPC6 promoter (*p* < 0.0001, Figures 8A,B). Similarly, we observed that the overexpression of PPARγ promoted the binding of the PPARγ to the TRPC6 promoter (*p* < 0.0001, at sites 1 and 2) but decreased the binding of the p65 to the TRPC6 (*p* < 0.0001, at sites 1 and 3, Figures 9A–D). All of these changes were reversed after the inhibition of the PPARγ gene. Furthermore, the hypoxia treatment increased the binding of the p65 to the promoter of the TRPC6 gene but decreased the binding of PPARγ protein to the TRPC6 promoter. These results suggested that the binding activity of PPARγ and p65 to the TRPC6 promoter was hypoxia dependent. Moreover, there was a competitive binding activity between PPARγ and p65 to the TRPC6 promoter in the hPASMCs. This mechanism is illustrated in Figure 10.

## Discussion

Our study demonstrated that the upregulation or downregulation of the p65 gene under the hypoxic condition promoted invasion, migration, proliferation, and Ca^2+^ influx (only overexpression) in the hPASMCs. Furthermore, we found that NF-κB/p65 binds to the TRPC6 promoter under the hypoxic conditions and PPARγ to the TRPC6 promoter under normal conditions. Under hypoxic conditions, NF-κB/p65 competed with PPARγ for TRPC6, resulting in the migration and proliferation of hPASMCs. These molecular and pathological changes may eventually result in the development of PAH in clinical settings.

When PAH develops or aggregates, an imbalance between vasoconstriction and vasodilation occurs, altering the related [Ca^2+^]i level and activity of cation channels (Guo et al., 2017; He et al., 2018; Tran et al., 2020), on which our study was focused. PAH is characterized pathologically by increased [Ca^2+^]i as well as vascular tissue growth and remodeling. Both TRPC1 and TRPC6 are members of the SOCC, which controls the SOCE and Ca^2+^ influx (Wang et al., 2006; Lu et al., 2008; Xu et al., 2014; Zhang Y. et al., 2014). The increased cell proliferation and growth in the aortas or PASMCs have been identified as a characteristic feature of PAH (Suresh et al., 2018). The [Ca^2+^]i level is associated with the cell proliferation and autophagy in a variety of cell types, including hPASMCs (Ogawa et al., 2008; Hamid and Newman, 2009; Yu et al., 2009; Du et al., 2017; Malczyk et al., 2017; Xu et al., 2017; Hodeify et al., 2018; Ji et al., 2018; Song et al., 2018; Zhu et al., 2018). Previous studies showed that hypoxia upregulated cation channels [including SOCE, TRPC6, and receptor-operated Ca^2+^ entry (ROCE)] and increased cytosolic [Ca^2+^] in PASMCs (Guo et al., 2017; He et al., 2018). Furthermore, hypoxia-induced mitochondrial dysfunction may be responsible for PAH (Belecanech et al., 2020). This study confirmed that hypoxia increased the level of TRPC6 and [Ca^2+^]i in hPASMCs, establishing a link between hypoxic condition and PAH.

Ca^2+^ influx-mediated cell proliferation is associated with many signaling pathways, including the NF-κB, cAMP response element, inositol 1,4,5-trisphosphate receptors (IP_3_Rs), and ryanodine receptors (RyR) (Lu et al., 2013; Hodeify et al., 2018). The proliferative role of IP_3_Rs has been widely recognized in cancer cells (Mound et al., 2017; Rezuchova et al., 2019) and mouse PASMCs (Shibata et al., 2019). The inhibition of type 2 IP_3_R inhibited the progression of PAH in mouse PASMCs through inhibiting SOCE signaling and inducing apoptosis. On the other hand, the activation of the NF-κB signaling pathway is associated with the development of PAH (Han and Logsdon, 2000; Jin et al., 2014; Li et al., 2019; Jang et al., 2021). Our study showed that the expression of NF-κB/p65 was increased by hypoxia treatment and was associated with different cell behaviors in PASMCs (Figure 10). Furthermore, the upregulation of p65 induced the expression of TRPC1/6 and increased Ca^2+^ influx into hPASMCs. These findings indicate that the expression of the NF-κB/p65 is related to the development of PAH via regulating the TRPC1/6 and SOCE in the hPASMCs. Therefore, the inhibition of NF-κB/p65 might be a potential therapeutic target for the treatment of PAH.

It is worth mentioning that there was a competitive relationship between NF-κB/p65 and PPARγ proteins for TRPC6. We identified and confirmed the binding sites of NF-κB/p65 and PPARγ proteins in the promoter regions of the TRPC6 gene. In our earlier research, we demonstrated that hypoxia-induced inhibition of PPARγ alleviates PAH (Wang et al., 2015), as well as the therapeutic action of PPARγ on PAH, by controlling the downstream TRPC1/6, SOCE, and hypoxia-induced PASMC proliferation (Gong et al., 2011; Zhang D. et al., 2014; Wang et al., 2015). Furthermore, Zhang et al., showed that activating PPARγ inhibited the proliferation of PASMCs and benefited PAH. In contrast to our earlier findings of downregulation of PPARγ in response to hypoxia, this study found that the hypoxia treatment had no significant effect on the expression of PPARγ in hPASMCs, but it decreased the binding activity of PPARγ to TRPC6. This difference may be due to the duration of exposure to hypoxia (72 h in this study vs. 12 h in the previous study), which means this phenomenon could be observed under chronic stimulation. Moreover, our study demonstrated that the inhibition of NF-κB/p65 increased the binding of the PPARγ protein to the TRPC1/6 promoter, which subsequently suppressed the TRPC1/6 activity and the SOCC and SOCE, which was consistent with our previous findings (Wang et al., 2015).

The most interesting finding was that NF-κB/p65 competed with PPARγ for TRPC6 in regulating TRPC6-mediated PAH. Many studies showed that NF-κB competes with PPARγ in inflammation and myocardial ischemia–reperfusion injury (Eisele et al., 2013; Scirpo et al., 2015; Liu et al., 2016; Zhang et al., 2016). NF-κB is a pro-inflammatory transcription factor that regulates inflammatory mediators and inflammation-mediated organ damage (El-Sheikh et al., 2017; Kurosawa et al., 2019). NF-κB links hypoxia and innate immunity through transcriptional regulation of HIF-1α (Rius et al., 2008; Remels et al., 2015; D’Ignazio et al., 2016; Santos and Andrade Júnior, 2017), an inhibitor of PPARγ (Wang et al., 2013b). NF-κB-mediated HIF-1α, or vice versa, has been extensively documented in various conditions, including malignant lymphoma and immune responses (Rius et al., 2008; Van Uden et al., 2008; Qiao et al., 2010; Remels et al., 2015; D’Ignazio et al., 2016). Our previous study revealed that HIF-1α suppressed PPARγ by acting upstream of it (Wang et al., 2015). These findings show that the interaction between NF-κB and PPARγ is mediated by HIF-1α (Figure 10). Furthermore, in this study, we observed that hypoxia increased the expression of p65 proteins binding to the TRPC6 promoter region, which was further enhanced by p65 overexpression. However, the overexpression of PPARγ had the opposite effect. These findings revealed that hypoxia-induced NF-κB/p65 competed with PPARγ for TRPC6 binding, resulting in increased TRPC1/6 activity, elevated SOCE and Ca^2+^ influx, increased hPASMC proliferation, and developed PAH.

There is a limitation to our study. The competitive binding of NF-κB, p65, and PPARγ on the TRPC6 promoter and their contrary effects on PAH were primarily investigated at the cellular level. Therefore, further investigations in the animal PAH model are expected to provide more solid evidence to support our findings.

## Conclusion

Our study confirmed that the higher expression level of the p65 gene in the hypoxic conditions promoted invasion, migration, and proliferation in the hPASMCs. However, the inhibition of the p65 gene under hypoxic conditions suppressed cell invasion, migration, and proliferation in the hPASMCs. Furthermore, the hypoxia-induced NF-κB/p65 competed with PPARγ in binding to the TRPC6 promoter. These findings showed that PPARγ and NF-κB/p65 have a competitive effect on TRPC6 activity by binding to the TRPC6 promoter region. This competitiveness is associated with oxygen and PAH development and may be mediated by HIF-1α. Although the molecular mechanisms behind the competitive effect of NF-κB/p65 and PPARγ are not clear and have to be thoroughly studied, this study provides novel and valuable evidence on the pathology of PAH.

## Data Availability Statement

The original contributions presented in the study are included in the article/supplementary material, further inquiries can be directed to the corresponding author.

## Author Contributions

All authors listed have made a substantial, direct, and intellectual contribution to the work, and approved it for publication.

## Conflict of Interest

The authors declare that the research was conducted in the absence of any commercial or financial relationships that could be construed as a potential conflict of interest.

## Publisher’s Note

All claims expressed in this article are solely those of the authors and do not necessarily represent those of their affiliated organizations, or those of the publisher, the editors and the reviewers. Any product that may be evaluated in this article, or claim that may be made by its manufacturer, is not guaranteed or endorsed by the publisher.

## Footnotes

1
https://www.genomatix.de/
